# Assessment of the Competence of Nurses in Taking Care of a Dying Patient in Poland

**DOI:** 10.1007/s13187-023-02284-x

**Published:** 2023-03-27

**Authors:** Anna Sadowska, Izabella Krzykwa, Bożena Czarkowska-Pączek

**Affiliations:** Department of Clinical Nursing, Medical University of Warsaw, Warsaw, Poland

**Keywords:** Nursing care, Dying patient, Palliative care, FATCOD-BP, Hybrid education

## Abstract

During nursing education, few practical hours are devoted to comprehensively preparing students to care for a dying patient. Contact with a dying patient is a key element of the job of every working nurse in the profession. Therefore, it is necessary to properly prepare nurses to care for a dying patient. This study aimed to assess the professional competence of nurses in caring for a dying patient and the factors that affect this preparation. This study involved 223 nurses during master’s degree in nursing at the Medical University of Warsaw, receiving either full-time education (group I, *N* = 121) or hybrid education (group II, *N* = 102). The study used the FATCOD-BP ((Frommelt Attitudes Toward the Care Of the Dying scale Form B, Polish version)) questionnaire (Cronbach’s alpha 0.75), an original questionnaire containing questions about feelings experienced during the SARS-CoV-2 virus pandemic and sociodemographic questions. FATCOD-BP for all groups was below average regarding caring for a dying patient (*M* = 109, SD = 11.68). Nurses pursuing full-time education were better prepared to care for a dying patient than were nurses pursuing hybrid education. Nurses who exhibited fear of their own deaths had a lower subjective level of preparation for caring for a dying patient. (1) Nurses are not sufficiently prepared to care for a dying patient. (2) The training of nurses should be provided in the form of in-patient education, and the methods of training should be modified by increasing the number of hours of practical and theoretical instruction in palliative care for a dying patient.

**Supplementary Information:**

The online version contains supplementary material available at 10.1007/s13187-023-02284-x.

## Introduction

Nursing care for a dying patient means cooperating not only with the patient but also with their family. Therefore, the nurse is obliged to their profession not only in the scope of the professional duties with which they have been entrusted but also in the social and communication scope, with both the patient and their relatives.

In Poland, a nursing studies education includes first-degree studies, i.e., bachelor’s degree studies (3 years), and second-degree studies, i.e., master’s degree studies (2 years). After completing bachelor’s degree studies in nursing, a student has the right to practice as a nurse and the opportunity to work. On the other hand, master’s degree students pursue additional education and are not required to work as nurses. During bachelor’s degree studies in nursing, students are educated for 80 h in palliative care consisting of practical teaching methods [1]. This subject is not continued in master’s degree studies. Therefore, few practical hours are available during the study period to comprehensively prepare for caring for a dying patient. Contact with a dying patient is an inseparable element of the responsibilities of every working nurse in the profession. Epidemiological data indicate that Polish society will age [2] and the need for palliative care will increase. Therefore, it is necessary to properly prepare nurses to care for a dying patient.

In Poland, on 20 March 2020, the SARS-CoV-2 virus pandemic was announced. It changed the way in which the first-degree and second-degree studies of nursing students were conducted (hybrid education) [3] and led many nursing students to realize that the profession of nursing can be difficult and dangerous to their health and life [4]. Advanced information technology has enabled the continuation of education via stationary education in the form of hybrid education, i.e., the implementation of educational methods in the form of stationary and remote classes depending on the threat that the virus posed. The pandemic also showed that contact with a dying patient is an integral part of the nursing profession and that this contact cannot be avoided in professional work, regardless of additional specializations or the department in which one works. Therefore, the aim is to assess the professional competence of nurses in caring for a dying patient and the factors that affect this preparation.

## Material and Methods

### Study Design

The study design is descriptive and cross-sectional to achieve the intended purpose. Data were collected using paper–pencil in December 2018 and using paper–pencil and online in e-mails from January to April 2021. The research group was divided into two groups: nurses who participated in full-time education (December 2018) (group I) and nurses who participated in hybrid education (January–April 2021) (group II).

### Sample and Setting

A convenience sample was used in the research. The participants were novice nurses according to Patricia Benner’s model. The criteria for inclusion in the study group were nurses who were pursuing master’s degree studies in nursing at the Medical University of Warsaw, who consented to participate in the study and who did answer all the questions included in the survey. The criteria for exclusion from the study group were nurses who were not pursuing master’s degree studies in nursing at the Medical University of Warsaw, who did not consent to participate in the study, and who did not answer all the questions included in the survey.

### Instrument

#### General Sample Characteristics

Sociodemographic data on age, gender, active occupation, and experience in caring for a dying family member were collected using a self-assessment questionnaire.

#### Attitude Toward Dying

The attitude toward dying among the respondents was examined using the standardized questionnaire FATCOD-BP (Frommelt Attitudes Toward the Care Of the Dying scale Form B, Polish version). The questionnaire contains 30 single-choice statements using a Likert scale from 1 to 5 (1 = strongly disagree; 2 = disagree; 3 = don’t know; 4 = agree; 5 = strongly agree). The group of 20 statements refers to the respondent’s attitude to the dying person, and the remaining 10 statements refer to the patient’s family. The scale includes 13 statements about a positive attitude, and 17 statements about a negative attitude. Cronbach’s alpha coefficient is 0.725.

#### SARS-CoV-2

Data on feeling fear for oneself, a family member or patient, and professional contacts during the SARS-CoV-2 pandemic were collected using a proprietary questionnaire containing 9 closed questions.

### Ethical Considerations

The study was conducted in accordance with the Declaration of Helsinki and approved by the institutional review board of the authors’ affiliated university (No. AKBE/226/2020). All respondents were informed about the purpose of the study and the principles of anonymity and voluntary participation. The fulfillment of the criteria for participation in the study and full completion of the questionnaire were considered consent to participate in the study.

### Data Analysis

Statistical analyses were performed using IBM SPSS Statistics 26.0. Basic descriptive statistics were generated using percent, median, mean, standard deviation, skew, kurtosis, and range (minimum–maximum). To detect the normal distribution, the Kolmogorov–Smirnov test was used. It showed no statistical significance between the groups. It also showed that FATCOD-BP both in the whole group and in group I and group II followed the normal distribution. To compare the two groups in terms of FATCOD-BP, analysis was performed using the *t* test for independent samples or the Mann–Whitney *U* test. The Kruskal–Wallis *H* test was performed to compare more than two groups. A Spearman correlation analysis was performed to establish the relationship between FATCOD-BP and age. Then, using moderation analyses, the researchers checked on whether caring for a dying family member in the past, fear of one’s own death, or the death of a relative or patient moderated the subjective assessment of the preparation of novice nurses to care for a dying patient. The level of significance was *α* = 0.05.

## Results

### Characteristics of the Investigated Group

In total, 223 nurses who were pursuing master’s degrees in nursing at the Medical University of Warsaw and who were eligible to practice as nurses participated in the study. Group I (*N* = 121) consisted of nurses pursuing full-time education. Group II (*N* = 102) consisted of nurses pursuing hybrid education. The vast majority of members of group I and group II were women who were in the youngest age range, professionally active, and caring for a dying family member. Detailed data are presented in Table 1.

**Table 1:** Characteristics of the study group

	All group (*N* = 223)			Group I (*N* = 121)		Group II (*N* = 102)	
	*N*	Mean	%	*N*	%	*N*	%
Age							
21–23	174	87	78.0	101	83.5	73	71.6
24–26	22	11	9.9	3	2.5	19	18.6
27–30	7	3.5	3.1	5	4.1	2	2.0
> 30	20	10	9.0	12	9.9	8	7.8
Sex							
Female	219	109.5	98.2	118	97.5	101	99.0
Male	4	2	1.8	3	2.5	1	1.0
Professional activity							
Yes	175	87.5	78.5	97	80.2	78	76.5
No	48	24	21.5	24	19.8	24	23.5
Caring for a dying family member							
Yes	92	46	41	49	40.5	43	42.2
No	131	65.5	59	72	59.5	59	57.8

### Basic Descriptive Statistics for FATCOD-BP and Comparison of Group I and Group II in Terms of Attitudes Toward Caring for a Dying Patient

The results showed that the mean of FATCOD-BP for all groups was indifferent (*M* = 109, SD = 11.68). Group I and group II were also indifferent (*M* = 113.42 [SD = 10.09] for group I and *M* = 103.75 [SD = 11.30] for group II). To compare attitudes toward the care of a dying patient among those surveyed in group I and group II, a *t* test analysis was performed for independent samples. The analysis showed significant differences in the level of attitude (*t*(221) = 6.75; *p* < 0.001; *d* = 0.91; 95% CI [6.84; 12.49]). Respondents in group I showed a more positive attitude than those in group II.

### FATCOD-BP and the Assessment of Preparation for the Care of a Dying Patient

The analysis was carried out using the Kruskal–Wallis *H* test to check the differences in the FATCOD-BP level depending on the care of a dying patient for group I. The analysis showed significant differences between the groups (*H* (3) = 10.58; *p* = 0.014). Detailed analysis of the results using the Dunn post hoc test with a Bonferroni significance level adjustment showed that nurses who do not work with a dying patient and who do not plan to engage in such work in the future display a significantly lower level of conviction and positive feelings toward caring for a dying patient than those planning to care for a dying patient in the future (*p* = 0.027).

A Spearman correlation analysis for group II was performed for the relationship between FATCOD-BP and the assessment of preparation for caring for a dying patient. The analysis showed no relationship between the variables (*r*s = 0.13; *p* = 0.178).

### Correlations Between FATCOD-BP in the Whole Group, Group I, and Group II and Sociodemographic Variables

#### Caring for a Dying Family Member/Close Person

To compare the attitudes of nurses who cared for a dying family member or a close person, a *t* test analysis was performed for independent samples. For the whole group and group II, the differences between the groups were statistically insignificant (*p* = 0.219 and *p* = 0.270, respectively). The analysis showed that the attitudes of nurses caring for a dying family member/relative were more positive toward caring for a dying patient than were the attitudes of those from group I who were not caring for a loved one (*t*(221) = 3.46; *p* < 0.001; *d* = 0.64; 95% CI [2.64; 9.73]). The differences between the groups were statistically insignificant for group II.

#### Age

To establish a relationship between age and attitude, a Spearman correlation analysis was performed. The analysis showed no significant relationship between FATCOD-BP in all groups and ages (*r*s = 0.05; *p* = 0.473).

#### Sex

To compare men and women, an analysis was carried out using the Mann–Whitney *U* test for the entire group. For the whole group, the analysis did not reveal any significant sex-related differences (*Z* =  − 1.45; *p* = 0.147; *r* = 0.10). The analysis did not reveal any significant sex-related differences for group I (*Z* =  − 0.12; *p* = 0.907; *r* = 0.01). For group II, analyses were not carried out because there was only one man in this group.

#### Professional Activity

The Mann–Whitney *U* test was used to compare the attitudes of the executors and non-practicing nurses. The analysis did not show any significant differences in the attitudes of nurses working in the profession or not working in the profession for the whole group (*Z* =  − 1.76; *p* = 0.079; *r* = 0.12), group I (*Z* =  − 0.87; *p* = 0.383; *r* = 0.08), and group II (*Z* =  − 1.48; *p* = 0.140; *r* = 0.15).

### FATCOD-BP and Fear of Death in Group II

Through use of the *t* test for independent trials, the attitude toward caring for a dying patient was compared depending on the subjective fear of one’s own death and the death of the patient in group II.

There were no statistically significant differences between nurses who felt subjective fear of their own deaths (*M* = 102.72; SD = 9.23) and who did not feel subjective fear of their own deaths (*M* = 104.61; SD = 12.77) relative to attitudes toward caring for a dying patient (*t*(100) =  − 1.94; *p* = 0.055; *d* = 0.54; 95% CI [− 12.23; 0.14]).

Differences in the assessment of the subjective fear of death of the patient depending on the attitude toward caring for the dying patient were also irrelevant (*t*(100) =  − 0.03; *p* = 0.972; *d* = 0.01; 95% CI [− 4.54; 4.39]).

Those who felt a subjective fear of the patient’s death showed an indifferent attitude toward caring for the dying patient (*M* = 103.80; SD = 11.33), while those who were not afraid of the patient’s death showed a comparable attitude toward caring for a dying patient (*M* = 103.72; SD = 11.38).

A significant difference in the assessment of attitudes toward caring for a dying patient arose in groups distinguished by the fear of death of a loved one (*Z* = 02.08; *p* = 0.037; *r* = 0.21). Nurses who were afraid of the death of a loved one (*M* = 104.64; SD = 11.46) showed a more positive attitude toward caring for a dying patient than nurses who were not afraid of the death of a loved one (*M* = 98.60; SD = 9.01).

### Predictors of Subjective Assessment of Preparation for the Care of a Dying Patient

To determine which of the variables were significant predictors of the subjective assessment of preparation for the care of a dying patient, an ordinal regression analysis was performed. The following predictors were included in the model: caring for a dying family member, fear of one’s own death, and the death of a loved one or a patient. The analysis showed that the model was well fitted to the data (*χ*^2^ (7) = 95.55; *p* = 0.030), and explained 14.1% of the variance of the dependent variable (Cox and Snell *R*^2^ = 0.141). The analysis showed that nurses who show fear of their own death have a lower subjective level of preparedness to care for a dying patient compared to nurses who do not show a fear of dying. This variable was the only significant predictor of the subjective assessment of the preparation for caring for a dying patient. The other variables were not significant predictors. The results are summarized in Table 2.

**Table 2:** Ordinal regression coefficients for the model explaining the subjective assessment of preparation for caring for a dying patient

Predictors	*B*	SE	*Z*	*p*	95% CI	
					LL	UL
Not enough^a^	− 1.03	0.38	7.35	0.007	− 1.77	− 0.29
Enough^a^	1.33	0.39	11.43	0.001	0.56	2.10
Caring for a dying person (yes)^b^	0.16	0.39	0.16	0.688	− 0.61	0.93
Fear of your own death (no^c^)	1.06	0.52	4.11	0.043	0.04	2.09
Fear of your own death (I don’t know^c^)	− 0.34	0.52	0.42	0.515	− 1.35	0.67
Fear of a loved one dying (no^c^)	1.57	0.98	2.55	0.111	− 0.36	3.49
Fear of a loved one dying (I don’t know^c^)	0.00	0.76	0.00	0.996	− 1.49	1.50
Fear of a patient dying (no^c^)	− 0.86	0.52	2.77	0.096	− 1.87	0.15
Fear of a patient dying (I don’t know^c^)	− 0.49	0.50	1.00	0.318	− 1.47	0.48

^a^The reference category is the grade “Good”

^b^The reference category is “Yes”

^c^The answer category is “No”

### Caring for a Dying Family Member vs. Working in a Hospice

To establish the relationship between caring for a dying family member vs. working in a hospice, an analysis of the *χ*^2^ independence test was performed. The analysis showed no relationship between the variables (*χ*^2^ (3) = 6.41; *p* = 0.093; *V* = 0.23). The results are summarized in Table 3.

**Table 3:** Analysis of the frequency of work in a hospice depending on the care of a dying family member/close person

Caring for a dying family member/close person	Work in hospice, *n* (%)			
	At the moment, I work with dying people	Yes, in the future, I plan to work in caring for the dying	No	I don’t know
Yes	5 (50.0)	5 (83.3)	28 (34.6)	11 (45.8)
No	5 (50.0)	1 (16.7)	53 (65.4)	13 (54.2)

### Nurses’ Attitude Toward Caring for Dying People During Hybrid Education

Based on the obtained results, the attitude of nurses from group II toward caring for a dying patient during hybrid education was determined. The surveyed nurses answered questions related to the SARS-CoV-2 epidemic. To determine whether attitudes toward caring for a dying patient were different depending on the answers to the questions related to SARS-CoV-2, analyses were performed using the Kruskal–Wallis *H* test. The results of the analyses are presented in Table 4.

**Table 4:** Comparison of nurses’ attitudes toward caring for a dying patient (FATCOD-BP) in relation to caring for people dying in connection to the SARS-CoV-2 epidemic during hybrid education

Has the current epidemic situation with SARS-CoV-2 made you:	No		I don’t know		Yes		*H* (2)	*p* value	*η*^2^
	Me	IQR^a^	Me	IQR^a^	Me	IQR^a^			
Afraid of your own death?	105.50	18.00	99.50	7.25	99.50	18.00	8.81	*0.012*	0.07
Afraid of your family members dying?	110.50	30.25	101.50	17.75	102.00	17.75	0.47	0.792	< 0.01
Afraid of your patients dying?	106.00	17.00	99.50	8.50	102.00	23.00	4.34	0.114	0.02
Concerned about having personal contact with people from work?	107.00	18.00	99.00	8.50	96.00	9.00	15.1	*0.001*	0.13
Concerned about having personal contact with people outside of work who are on the medical staff?	107.00	1.00	99.50	10.00	97.00	15.00	13.3	*0.001*	0.11
Concerned about your own health?	105.50	18.50	102.00	18.50	100.50	15.50	5.13	0.077	0.03
Concerned about the health of your family members?	104.00	26.00	105.00	6.00	102.00	15.50	0.09	0.958	< 0.01
More stressed while performing nursing activities with your patients?	110.50	23.00	100.00	12.00	101.00	13.00	7.71	*0.021*	0.06
Experience a significant impact on your knowledge and experience during master’s degree studies?	102.00	17.00	101.50	15.50	103.00	16.00	0.17	0.917	< 0.01

*Me* median

^a^Interquartile stretch mark

Detailed analysis of the results via the Dunn post hoc test with a Bonferroni significance level adjustment showed significant differences in attitudes between nurses who provided the answers *I don’t know* and *no* (*p* = 0.030) in terms of fear of their own deaths. Nurses who answered did *not* have a more positive attitude than nurses who answered *I do not know*. Respondents who provided the answer *not* to the question about the fear of establishing direct contact with another healthcare worker in the workplace showed a more positive attitude than those who provided the answer *I do not know* (*p* = 0.047) or *yes* (*p* = 0.001). Respondents who provided the answer *not* to the question about the fear of establishing direct contact with another healthcare worker outside the workplace showed a more positive attitude than those who provided the answer *I do not know* (*p* = 0.018) or *yes* (*p* = 0.007). The nurses who answered *no* to the question about experiencing a higher level of stress during nursing/nursing activities toward the patient showed a more positive attitude than those who provided the answer *I do not know* (*p* = 0.045) or *yes* (*p* = 0.040). For the remaining questions related to the SARS-CoV-2 virus, the differences in the level of attitude were statistically insignificant.

## Discussion

In the nursing profession, work involves providing medical care not only to chronically ill patients but also to dying patients. The results show that nurses have received below-average preparation in caring for a dying patient. This result might be due to the low number of hours of practical and theoretical classes in the field of palliative care and ineffective methods of training nurses in this area. In Poland, during the education of nurses at the second degree, there is no teaching in the field of ethics toward the death of a sick person and psychology, which seem necessary in this field. Muliira et al. [5] indicate that nursing students are moderately prepared to provide palliative care. Similar results were obtained by Huriah et al. [6]. In addition, research conducted on the FATCOD-B questionnaire by Hao et al. [7] before and after further training showed that additional nurse training increases the level of preparation for caring for a dying patient. Therefore, the scope of practical and theoretical knowledge of nurses should be increased through simulations, instructional videos, and training in psychology; the ethics of the nursing profession; and communication with the patient and their family.

Our research also showed that nurses in group I were more prepared to care for a dying patient than were nurses in group II. This result might be due to the introduction of distance learning and hybrid learning from 2020 through the period of the greatest impact of the SARS-CoV-2 virus disease. The literature contains the results of studies by other authors, which indicate that distance learning raised concerns among nursing students about achieving their intended professional competencies [8, 9]. The results of our own and other authors’ research suggest that the distance education method is insufficient for nurses. Therefore, it is necessary not only to conduct classes in a stationary form but also to increase the number of practical hours at the bedside of a dying patient through practice during teaching as well as outside it through training and vocational refresher courses in this field.

Knowing and understanding the needs of patients is very important to providing comprehensive care. The results of our own research suggest that the lack of professional experience among nurses and the lack of willingness to care for a dying patient in the future have a negative impact on the level of preparation for care for this group of patients. This result might stem from the insufficient number of practical and theoretical classes available in palliative care during academic education, which would enable comprehensive preparation in the mental and physical aspects of caring for a dying patient and encouraging nurses to work with such patients in the future. Jeznach et al. [10] showed that medical students, including nurses, despite having contact with sick and dying people, are not comprehensively prepared for a patient’s death. On the other hand, studies by other authors indicate that longer work experience among nurses significantly heightens empathy toward dying people [11]. In addition, the scientific research of Niedojad et al. [12] shows that the length of service in the nursing profession has a positive impact on the need to provide decent conditions for the patient to die [12]. The above research results suggest that the scope of academic education should change in terms of teaching methods and by increasing the number of hours in the field during which students engage in practical care of a dying patient. This will prepare nurses for having physical and mental contact with such a group of patients. It can be assumed that nurses who have prepared comprehensively to work with a dying patient will be more likely to work in a hospice, where there is a great demand for nursing staff prepared to provide palliative care as well as care for the elderly, due to an aging population.

This research found that nurses in group I who had experience caring for a dying loved one or family member experienced a positive impact on attitudes toward caring for a dying patient. In the scientific literature, no research results were found with which to compare our own results, while the results of our own research suggest that nurses who cared for dying relatives became acquainted with the process of such care, which prepared them for patient care. The study did not show a static significance for group II. The impact on this result might have been due to a sudden and dynamic pandemic phenomenon, in which nurses in group II repeatedly experienced the phenomenon of dying among relatives and feared for their lives, unlike nurses in group I.

According to psychologists, the fear of dying is one of the strongest emotions, even though it is an integral part of the human biological process [13]. Our research has shown that the manifestation of subjective fear among nurses regarding the death of a loved one causes a more positive attitude toward caring for a dying patient. This result might indicate that anxiety as a mental state of danger for a loved one motivates a positive attitude toward caring for a dying patient. No scientific studies have been found in the literature to compare with our own results, but the fact is the stressor of anxiety has a motivating effect on taking action to solve problems [14]. In addition, nurses who fear a loved one’s death identify themselves by empathizing with the feelings of the families of dying people, which can motivate them to maintain a more positive attitude toward caring for a sick or dying person. Also, our research has revealed that, among nurses, showing fear of their own deaths subjectively lowers the level of preparation for caring for a dying patient. This result might be due to the lack of emotional resistance to the biological phenomenon of dying, which could affect the quality of care for a dying patient. Research by Niedojad et al. [12] indicates that nurses are afraid of their own deaths [12]. Preliminary scientific reports indicate that nurses are willing to follow psychologists’ advice [15]. In addition, research by Sleziona and Krzyżanowski [16] indicates that a patient’s death lowers nurses’ fear of their own deaths [16]. It can be assumed that comprehensive preparation among nurses to practice in the field of psychology and deal with the fear of their own deaths—through education and the support of psychologists in the workplace—reduces nurses’ level of fear of their own deaths, which will also enable comprehensive preparation for caring for a dying patient.

In the extraordinary time of the pandemic, which affected the whole world, nurses stood on the front lines of the fight against the disease. This phenomenon has had many psychological consequences for healthcare workers [17]. Our results show that nurses who participated in hybrid education and who had no fear of their own deaths due to SARS-CoV-2 were not afraid of coming in contact with other healthcare workers either at work or outside the workplace and did not experience an increased feeling of stress while performing nursing duties for patients during the pandemic. They had higher results in terms of preparing for the care of a dying patient. This result might be due to mental immunity against working in conditions of infection and having adequate knowledge in the field of health protection and protective measures against infection at work. In addition, the result might have been influenced by the fact that the respondents were young and had little work experience. Scientific studies by other authors indicate that, during the pandemic, nurses experienced a fear of infection, death, and the loss of a loved one, as well as of contact with people who might be infected [18]. Moreover, the perception of infection risk among Polish nurses was a predictor of perceived stress [17]. It can be concluded that nurses who accepted the occupational risk of the accompanying pandemic and the consequences of this phenomenon were more positive about caring for a dying patient. This fact might be the result of the receipt of appropriate training in the workplace, the protection of precautions in the work of medical personnel with regard to the conditions of the pandemic, and the acceptance of increased human mortality due to the pandemic. It can be concluded that appropriate training in epidemiology and occupational safety rules and the procurement of personal protective equipment (masks, goggles, disposable aprons, disinfectants) will reduce the level of stress and anxiety among medical workers. It should be borne in mind that in Poland, the phenomenon of the pandemic is under control but medical staff must receive comprehensive education about the emission of infection and be secure in the workplace out of concern for their own health as well as that of their patients and loved ones.

In conclusion, nurses in Poland are not well prepared to care for a dying patient. Action should be taken to increase the level of preparation of nurses in this area, i.e., organize meetings or simulations with the patient, provide instructional videos and training in psychology, and hone communication with the patient and their family. In addition, nursing staff should be encouraged to participate in training and further education in palliative care and to seek psychological help in the workplace. There is also a need to increase the number of practical and theoretical hours in palliative care and meetings with a psychologist during nursing students’ education so that nurses can comprehensively learn how to help a dying patient. Additionally, it is important to broaden education in the field of epidemiology and the occupational safety of medical workers, including nurses, and to provide as much personal protective equipment as possible, as the phenomenon of epidemics can occur again in any country, including Poland.

## Limitations

This study has a few limitations. One of them is the age of the respondents. They are young nurses who have little experience in their professional work and in working with a dying patient. Most of the respondents were women, which might affect the results of the research due to the psychological characteristics of the female sex. Moreover, the nurses participating in this study were graduates of only one medical university in Poland and were completing their master’s degrees in nursing.

## Conclusions

1. Nurses are not sufficiently prepared to care for a dying patient.
2. The training of nurses should be provided in the form of in-patient education, and the methods of training should be modified by increasing the number of hours of practical and theoretical instruction in palliative care for a dying patient.
3. Additional elements of training for nurses in psychology, ethics, and interpersonal contact with the dying patient and their family should be introduced.
4. Comprehensive training of nurses on the emission of infection should be introduced.
5. Maximum personal protection for nurses and comprehensive training in epidemiology in the workplace should be introduced.

### Supplementary Information

Below is the link to the electronic supplementary material.

Supplementary file1 (DOCX 25 KB)
