# Supplementary material for: Assessment of the Competence of Nurses in Taking Care of a Dying Patient in Poland
Source: J Cancer Educ. 2023 Mar 27;38(5):1471–8. doi: 10.1007/s13187-023-02284-x (PMC10042412; doi:10.1007/s13187-023-02284-x)
Supplement: Supplementary file 1 — Supplementary file1 (DOCX 25 KB) [file 13187_2023_2284_MOESM1_ESM.docx]

**SUPPLEMENTARY MATERIAL**

**Correlations between the FATCOD-BP results in the whole group, Group I, and Group II**

To compare the responses to individual FATCOD-BP questions for the whole group, Group I, and Group II,
a Mann-Whitney U test was performed. The analyses were carried out on the recorded items, which means that the higher the result, the more positive the attitude. Statistical analysis for the whole group showed that the highest median occurred in the case of questions 18 and 20. This, in turn, means that the respondents indicated the highest answers to these questions. Question 8 was rated the lowest; the median for this question was 2. Group I, compared to Group II, had a statistically significantly more positive attitude toward positive statements: 1, 2, 12, 22, 24, 25, 26, 27. The statistical analysis also showed statistical significance in the statements defining a negative attitude: 5, 6, 9, 11, 13, 14, 15, 17, 19, 28. Here, the results also show that Group I is more positive about caring for a dying patient than is Group II. The remaining statements showed no statistical significance for either Group I or Group II. The results of the analyses are presented in Table 5.

Table 5. Comparison of FATCOD-BP results for the whole group, Group I and Group II.

|  | All group | | Group I | | | Group II | | |  |  |  |
| --- | --- | --- | --- | --- | --- | --- | --- | --- | --- | --- | --- |
|  | *Me* | *IQR* | *M* | *Me* | *IQR* | *M* | *Me* | *IQR* | *Z* | *p* | *r* |
| 1. Giving care to the dying person is a worthwhile experience. | 4,00 | 1,00 | 128,07 | 4,00 | 1,00 | 92,93 | 4,00 | 1,25 | -4,34 | **<0,001** | 0,29 |
| 2. Death is not the worst thing that can happen to a person. | 4,00 | 2,00 | 119,84 | 4,00 | 2,50 | 102,70 | 3,00 | 2,00 | -2,03 | **0,042** | 0,14 |
| 3. I would be uncomfortable talking about impending death with the dying person. | 3,00 2,00 | | 117,89 | 3,00 | 2,00 | 103,99 | 2,50 | 1,00 | -1,67 | 0,094 | 0,11 |
| 4. Caring for the patient's family should continue throughout the period of grief and bereavement. | 4,00 2,00 | | 116,64 | 4,00 | 2,00 | 106,50 | 4,00 | 1,00 | -1,22 | 0,221 | 0,08 |
| 5. I would not want to care for a dying person. | 3,00 2,00 | | 119,22 | 3,00 | 2,00 | 102,42 | 3,00 | 2,00 | -2,00 | **0,045** | 0,13 |
| 6. The non-family care-givers should not be the one to talk about death with the dying person. | 4,00 2,00 | | 128,01 | 4,00 | 1,00 | 93,00 | 4,00 | 1,00 | -4,26 | **<0,001** | 0,29 |
| 7. The length of time required to give care to a dying person would frustrate me. | 3,00 2,00 | | 114,48 | 3,00 | 2,00 | 109,06 | 3,00 | 2,00 | -0,64 | 0,519 | 0,04 |
| 8. I would be upset when the dying person I was caring for, gave up hope of getting better. | 2,00 1,00 | | 117,91 | 2,00 | 1,00 | 104,99 | 2,00 | 1,00 | -1,58 | 0,115 | 0,11 |
| 9. It is difficult to form a close relationship with the dying person. | 3,00 2,00 | | 122,74 | 3,00 | 2,00 | 97,31 | 3,00 | 1,00 | -3,05 | **0,002** | 0,21 |
| 10. There are times when death is welcomed by the dying person. | 4,00 1,00 | | 117,34 | 4,00 | 1,00 | 103,60 | 4,00 | 1,00 | -1,76 | 0,078 | 0,12 |
| 11. When a patient asks, "Am I dying?", I think it is best to change the subject to something cheerful. | 4,00 1,00 | | 128,09 | 4,00 | 2,00 | 91,06 | 3,00 | 2,00 | -4,46 | **<0,001** | 0,30 |
| 12. The family should be involved in the physical care of the dying person if they want to. | 5,00 1,00 | | 127,53 | 5,00 | 1,00 | 92,64 | 4,00 | 1,00 | -4,54 | **<0,001** | 0,30 |
| 13. I would hope the person I'm caring for dies when I am not present. | 3,00 2,00 | | 121,50 | 3,00 | 1,00 | 99,74 | 3,00 | 2,00 | -2,62 | **0,009** | 0,18 |
| 14. I am afraid to become friends with a dying person. | 3,00 2,00 | | 127,33 | 3,50 | 1,00 | 92,87 | 3,00 | 1,25 | -4,09 | **<0,001** | 0,27 |
| 15. I would feel like running away when the person actually died. | 3,00 1,00 | | 127,13 | 4,00 | 2,00 | 93,12 | 3,00 | 2,00 | -4,06 | **<0,001** | 0,27 |
| 16. Families need emotional support to accept the behavior changes of the dying person. | 4,00 1,00 | | 115,29 | 5,00 | 1,00 | 108,10 | 4,00 | 1,00 | -0,93 | 0,352 | 0,06 |
| 17. As a patient nears death, the non-family care-giver should withdraw from his/her involvement with the patient. | 4,00 2,00 | | 120,33 | 4,00 | 2,00 | 102,12 | 4,00 | 1,00 | -2,23 | **0,026** | 0,15 |
| 18. Families should be concerned about helping their dying member make the best of his/her remaining life. | 5,00 1,00 | | 109,65 | 4,00 | 1,00 | 113,67 | 5,00 | 1,00 | -0,52 | 0,603 | 0,03 |
| 19. The dying person should not be allowed to make decisions about his/her physical care. | 4,00 2,00 | | 127,70 | 4,00 | 1,00 | 93,38 | 4,00 | 2,00 | -4,16 | **<0,001** | 0,28 |
| 20. Families should maintain as normal an environment as possible for their dying member. | 5,00 1,00 | | 113,37 | 5,00 | 1,00 | 110,38 | 5,00 | 1,00 | -0,39 | 0,700 | 0,03 |
| 21. It is beneficial for the dying person to verbalize his/her feelings. | 4,00 2,00 | | 114,55 | 4,00 | 2,00 | 108,98 | 4,00 | 1,25 | -0,68 | 0,498 | 0,05 |
| 22. Care should extend to the family of the dying person. | 4,00 1,00 | | 118,53 | 5,00 | 1,00 | 98,26 | 4,00 | 1,00 | -2,62 | **0,009** | 0,24 |
| 23. Care-givers should permit dying persons to have flexible visiting schedules. | 4,00 2,00 | | 116,27 | 4,00 | 2,00 | 105,89 | 4,00 | 2,00 | -1,26 | 0,208 | 0,12 |
| 24. The dying person and his/her family should be the in-charge decision makers. | 4,00 1,25 | | 122,00 | 4,00 | 2,00 | 99,15 | 4,00 | 1,00 | -2,79 | **0,005** | 0,25 |
| 25. Addiction to pain relieving medication should not be a concern when dealing with a dying person. | 4,00 2,00 | | 124,04 | 4,00 | 2,00 | 96,75 | 3,00 | 1,00 | -3,28 | **0,001** | 0,30 |
| 26. I would be uncomfortable if I entered the room of a terminally ill person and found him/her crying. | 3,00 2,00 | | 119,10 | 3,00 | 2,00 | 102,55 | 2,00 | 1,00 | -1,99 | **0,047** | 0,18 |
| 27. Dying persons should be given honest answers about their condition. | 4,00 1,00 | | 122,61 | 4,00 | 1,00 | 98,43 | 4,00 | 1,00 | -3,00 | **0,003** | 0,27 |
| 28. Educating families about death and dying is not a non-family care-givers responsibility. | 4,00 1,00 | | 119,51 | 4,00 | 2,00 | 102,08 | 4,00 | 1,00 | -2,10 | **0,036** | 0,14 |
| 29. Family members who stay close to a dying person often interfere with the professionals' job with the patient. | 3,00 2,00 | | 114,59 | 3,00 | 2,00 | 107,87 | 3,00 | 2,00 | -0,81 | 0,419 | 0,05 |
| 30. It is possible for non-family care-givers to help patients prepare for death. | 4,00 1,00 | | 118,18 | 4,00 | 1,00 | 103,64 | 4,00 | 1,00 | -1,91 | 0,056 | 0,13 |

M – mean/average; Me – Median; IQR – interquartile stretch mark; Z – standard score; p – p-value; r – correlation coefficient
